# The Concept of Psychotextiles; Interactions between Changing Patterns and the Human Visual Brain, by a Novel Composite SMART Fabric

**DOI:** 10.3390/ma13030725

**Published:** 2020-02-05

**Authors:** George K. Stylios, Meixuan Chen

**Affiliations:** Research Institute for Flexible Materials, Heriot-Watt University, Scottish Borders Campus, TD1 3HF, UK; michellemeixuanchen@gmail.com

**Keywords:** SMART pattern-changing fabric, pattern effect, visual response, visual brain, event-related potential (ERP), psychotextiles, art and design

## Abstract

A new SMART fabric concept is reported in which visual changes of the material are designed to influence different human emotions. This is achieved by developing a novel electrochromic composite yarn, knitted into pattern-changing fabrics, which has high response in temperature change and uniform contrast. The influence of these pattern-changing effects on the response of the human visual brain is investigated further by using event-related potential (ERP). Four SMART pattern-changing fabric pairs were used in this experiment. Each fabric presents two patterns interactively with different, but complementary or opposing, pattern attributes. 20 participants took part in the experiment, in which they were exposed to the patterns, while their visual brain activities were recorded. Comparisons of the three prominent ERP components; P1, N1, and P2 that correspond to the two patterns of each fabric have shown significant differences in the latency and amplitude of these components. These differences show that patterns and pattern-changing cause different visual impacts and that these changes influence our level of attention and processing effort. The study concludes that with the pattern changing ability of these thermochromic hybrid materials we can create designs with attributes that can directly manipulate user emotions, which we like to call ‘psychotextiles’. Our study also poses much wider questions of our image processing process in relation to design and art.

## 1. Introduction

Recent advances of SMART textiles enable researchers to explore new ways of interaction with users. There is increasing interest to construct SMART structures, systems and prototypes with tailor-made functionality and aesthetics, which can interact with user’s behaviour. Colour, pattern, and shape-changing effects of SMART fabrics are now capable for interaction with our emotional responses [1,2,3,4,5,6,7,8]. Most of these designs however focus on exploring SMART fabrics capability rather than designing them to actively influence any emotional user response. This study aims to investigate whether it is possible to pre-determine the pattern design of a novel SMART fabric with pattern-changing properties which would directly influence human visual response.

Studies of pattern perception have found that the shape and form of pattern can influence human visual response. Consecutive circles, radical lines, and stripes had less effect than checkboard, with the smaller check pattern having higher response [9,10,11,12]. A triangular pattern had an even higher response than a circle and a square [13]. Sharp corners in patterns had a quicker response than rounded corners [14,15]. Sharpness of patterns were also important; sharp patterns produce quicker visual response than blurred ones [12]. The same is true with symmetrical patterns were quicker in response than asymmetrical ones [16]. There is a clear indication therefore that patterns can affect our visual responses, but there is lack of extended studies for providing concrete evidence, and if there is, how can these effects be facilitated in new materials. Hence, in this study, we test the hypothesis that patterns may affect our emotions, we concentrate here at the start of the image process (visual brain) and we facilitate this study by developing a new composite material capable of pattern changing in a predetermined way. The yarn had to change the colour quickly and uniformly and also to be durable to withstand stresses and strains imposed during fabric making. This study is particularly interesting because this novel SMART fabric enables us to devise pairs of design changes of the same fabric, to test the hypothesis of switching on-off and altering a specific emotion. The question to why we have decided on the four fabric pairs reported here is because in our preliminary experiments [17] we found that these designs were most important to test our concept and without the influence of colour in the first instance.

Two pattern characteristics were investigated in the current study: repeating/non-repeating and weak/intense. Repeating patterns contain regularly repeating elements and have symmetrical and continuous features; in contrast, non-repeating patterns contain irregularly repeating elements and have asymmetrical and discontinuous features. Repeating patterns have been previously found to have more pleasant effect than non-repeating patterns in one of our studies [17]. Weak patterns are faint, light, and simple compared to intense patterns that are high in contrast, bold, and complex. According to literature, the symmetrical feature of repeating patterns may trigger an earlier visual response from viewers than non-repeating pattern, and the intense patterns could evoke a higher visual response than weak patterns.

We appreciate that this study cuts through two different disciplines; materials and neuroscience. It is necessary, like in most other fields, to work interdisciplinarily. Our aim is however a new material; a new SMART textile which will, for the first time be capable of influencing specific emotions by precisely designing attributes found from studies such as ours. Hence, we call this new class of SMART fabrics that are designed to influence specific emotions ‘psychotextiles’. The results of our visual brain study are reported here we are preparing follow on results from our complimentary study of the frontal part of our brain.

Human visual response can be measured from the activity of the visual brain, which is in the most posterior portion of the brain and is the centre for processing all visual information that is received through our eyes. Measurement of the visual brain activity provides an objective insight into an individual’s visual response to an external stimulation. The brain activity can be measured by a non-invasive electroencephalogram (EEG) technique.

**Figure 1:**
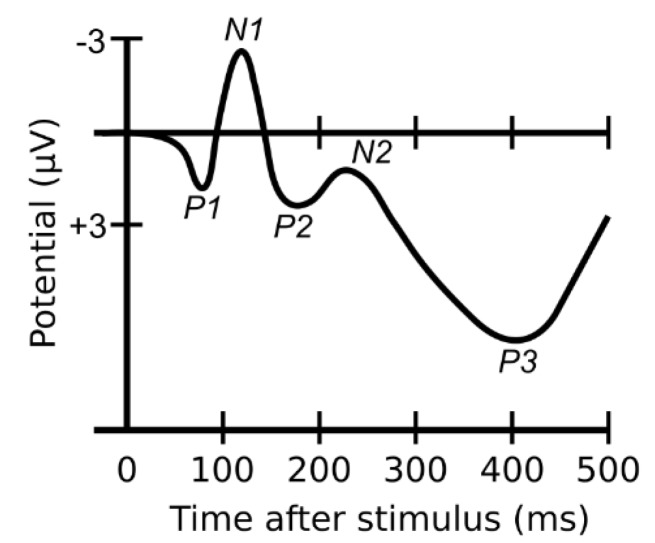
A typical ERP elicited by a visual stimulation.

Our brain consists of billions of neuron cells which work together to perform various functions. They operate on electrochemical transmission. Synchronised neurons produce electric potential (EP) in the brain and by attaching electrodes on the scalp this activity can be measured by amplification of these signals. We call EEG the waveform trace that is recorded from the EP over a known time period [18]. Therefore, the brain activity can be inspected by analysing the EEG traces. When responding to a stimulus, the electrical activity of the brain changes as soon as a response occurs. Some changes are large enough to be identified in the primary EEG trances, but some are rather small and concealed inside the unrelated spontaneous brain activities. In order to discriminate the electrical activity that is corresponding to the specific stimulus from the noise that is generated by unrelated activities, a signal averaging approach is used to increase the amplitude of the event related signal relative to noise, in which the specific stimulus has to be repeatedly presented or conducted for a number of trials, then the time-locked EEG signals corresponding to the stimulus are extracted from the continuous EEG record, aligned, and the amplitudes on each time point in the signal are then averaged. Connecting the averaged amplitude at each time point obtains a wave form, which is the event-related potential (ERP) that presents the discrete brain activity responding to a specific stimulus [19,20,21]. Figure 1 shows a typical ERP evoked by a visual stimulation. A sequence of prominent picks can be found in an evoked ERP, which are named by their negative (N) or positive (P) polarity. As seen in Figure 1, the component P1 is the first prominent positive peak; component N1 is the first prominent negative peak; accordingly, component P2 and P3 is the second and the third positive peaks. The occurrence, the amplitude and latency of the ERP component have been found varied depending on the parameter of the stimulus and on the subject’s psychological state.

The ERP measurement is relatively straightforward. It has been use in studies of stimulation effect on viewers’ response, such as perception of colour and pattern [22], affective processing [23], and facial expression processing [24]. In our study, viewers’ visual brain response to a pattern stimulus is measured by using the ERP technique and analysed on the evoked ERP components.

## 2. Experimental Methodology

### 2.1. Materials and Processing Methods

#### 2.1.1. Yarn

**Figure 2:**
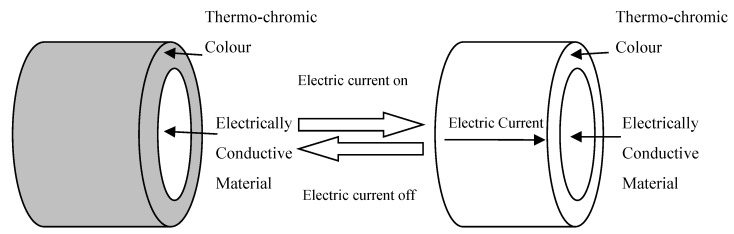
Conceptual model of the SMART colour-changing electrochromic yarn.

The pattern-changing effect of the fabrics is based on a novel composite electrochromic yarn that can change colour by changes of the electric current. As presented in Figure 2, the composite yarn consists of a core and outer sheath. The outer sheath consists of a thermochromic material that can change colour at a given temperature. Colour change is produced by heat being produced in the core of the yarn by electric current passing through it, rendering the changing of the colour on its thermochromic outer sheath. The temperature is dropped by stopping the electric current resulting in heat dissipation in the core of the yarn and causing the thermochromic outer sheath to go back to its original colour. Hence, colour can be changed in the yarn by passing a known electric current to the yarn. After experiments with different diameters, a core diameter of 0.10 mm silver-plated copper wire was chosen, which was pliable enough and thin and it could produce adequate heat.

**Figure 3:**
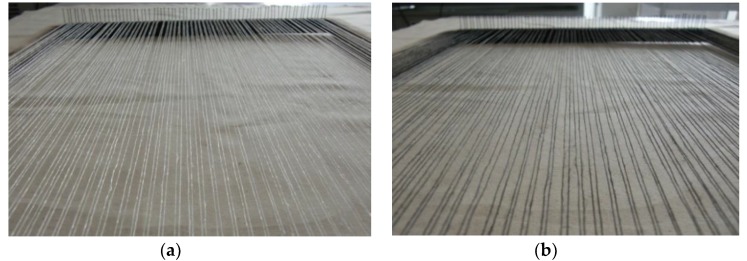
Composite copper/cotton yarn before; (**a**) and after; (**b**) colouration.

To increase its strength and pliability for fabric processing, it was blended with several yarns; 180 tex wool, 30 tex silk, 58 tex spun viscose, 20 tex cotton, using a Gemmill and Dunsmore hollow spindle fancy yarn machine, the conditions of which were optimised after preliminary investigations. Consequently, the yarn produced is a composite hybrid material of textile and copper, and as such non ordinary to use any thermochromic dye. After a number of experiments, it was decided to use a special thermochromic pigment (Chromazone’s Water based sprayable system 1510), suitable for metal surfaces, and we found for the first time that it was also successfully applied to textiles, since our composite yarn is composed of a metallic part in its core and a textile part in its sheath. The application of the pigment was done by Chromazon’s Sprayable System (LCR Hallcrest Ltd., Connah’s Quay, UK). The first step of this process is the preparation of the thermo-chromic colour system solution, which was made up of 55.9% clear lacquer, 24% thermo-chromic pigment, 1.1% adhesion promoter, and 19.0% water, mixed with a mechanical stirrer. The yarn was then wound onto a wooden frame around nails fixed in opposite sides and parallel to each other. The prepared solution was poured into the glass jar of an airbrush (Model 200 Airbrush, Badger Air-brush Company, Illinois, 9128 Belmont Av, Franklin Park, IL, USA) and evenly sprayed over the yarn. Spraying was repeated several times until the whole yarn was covered in black colour. Then, the prayed yarn set in the wooden frame was placed in a pre-heated oven of 100 °C for 5 min, for curing the thermo-chromic pigment. The yarn was left to cool down to room temperature, and then wound onto a yarn cone for knitting. Figure 3a,b shows the composite SMART yarn on the wooden frame before and after colouration.

**Figure 4:**
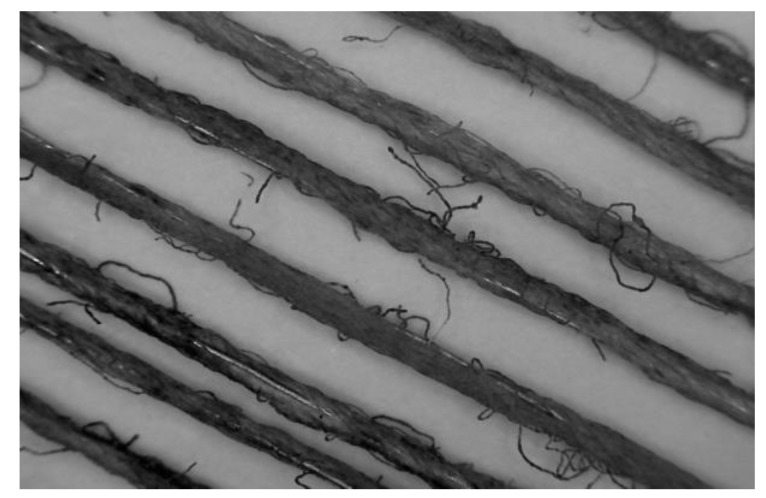
X35-times magnification coloured composite yarn.

**Figure 5:**
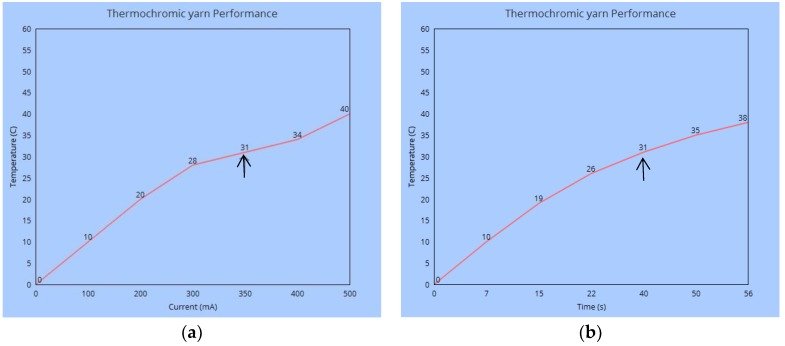
Performance of the composite thermochromic yarn; (**a**) temperature against current and (**b**) temperature against time. The arrows shows the colour change values.

Experiments were then carried out to find the most suitable cotton blend, by uniformly wrapping 4 metres of the yarn around a white cardboard in 20 °C and 65% RH conditioned room, and its colour change characteristics were investigated. The 20 tex cotton yarn was found to be the most suitable which has 9 Ohms electrical resistance and 0.3 A to 0.4 A optimum electric current, and is able to change from black to white in less than 40 s, Figure 4 shows a 35 times magnification image of the yarn and Figure 5a,b its thermochromic performance.

So we have established a hybrid composite yarn of cotton and copper which is now capable of colour changing at an activation temperature of 31 °C by heat being produced from the copper wire and triggers the thermochromic surface to change colour from black to colourless and reverse uniformly and in less than 40 s. Investigations into different knitting and woven processes that followed revealed that the fabric made on an 8-gauge Shima-Seiki SES 122S electronic knitting machine (SHIMA SEIKI Europe Ltd., Castle Donington, UK) had the best design reproduction and response to pattern change, at 0.5 metres per min speed, optimum tension, and with 0.7 mm stitch length.

Having established the fabric several preliminary investigations took place to determine the criteria of designs and their changes that will fulfil our hypothesis, and it was apparent that we should knit four paired fabric patterns. These four pairs were made and used for the brain/material experiments that followed.

#### 2.1.2. Pattern Changing Fabrics

**Figure 6:**
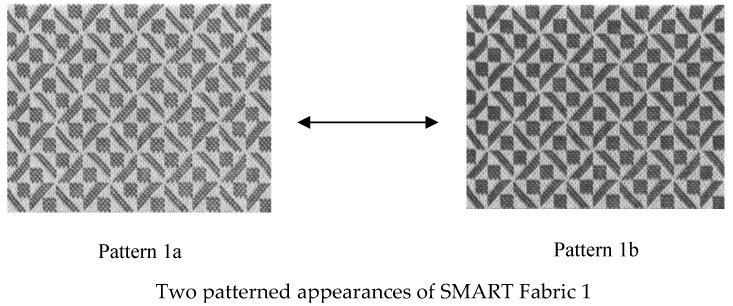

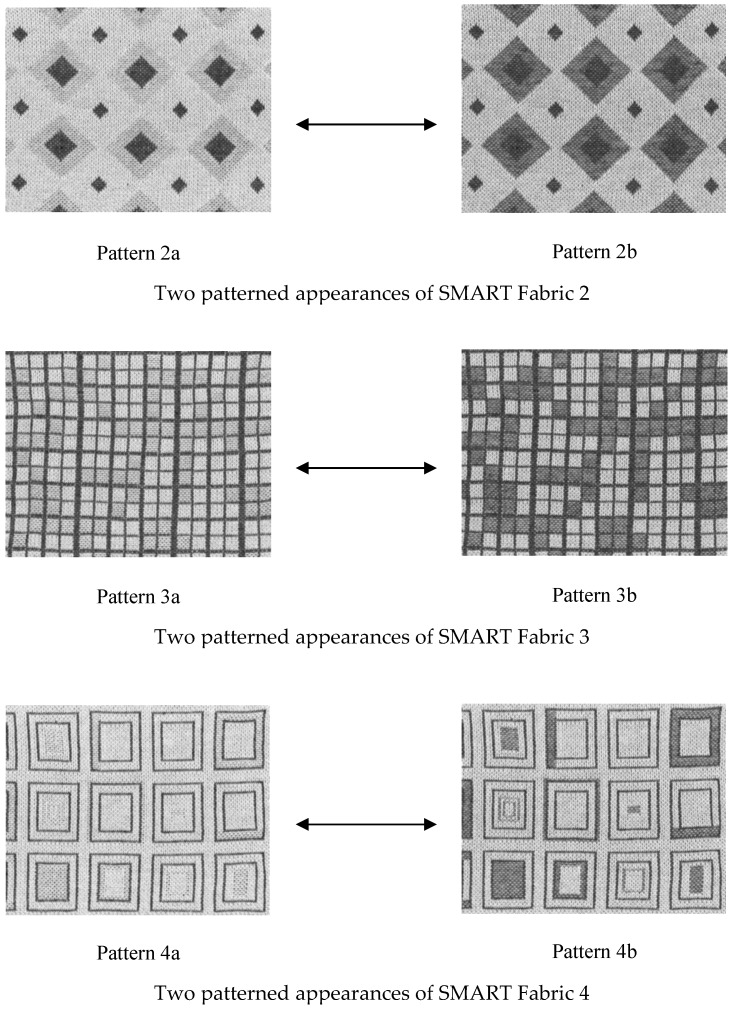
Four SMART pattern-changing fabrics investigated in current study.

These four pairs of patterns are shown in Figure 6. In order to test the response of our visual brain every pair of the four fabrics has the same design but subtle differences. Fabric 1a,b consists of geometric square and trapezoid small motifs but 1b are bolder (black and white more well defined—sharper) than 1a. Fabric pair 2, consists of diamond motifs both pairs are symmetrical but fabric 2b has larger diamonds making the symmetry more well defined than in the case of fabric 2a. Fabric pair 3 consists of small squares, with fabric 3a being symmetrical with squares of the same repeat, whilst fabric 3b produces random asymmetrical and irregular shapes with darker intensity. Fabric pair 4 consists of large square shapes, with fabric 4a being symmetrical and fabric 4b random asymmetric and irregular squares with darker intensity. Therefore, the predominant effect is geometry, asymmetry, faintness and boldness, intensity and irregularity, all in black and white and without the effect of colour so that there is no colour influence on our tests.

### 2.2. Material/Brain Interactions

Viewers’ visual response to a pattern stimulus is directly measured in the visual brain activity by using the ERP technique, so that the brain/pattern interaction can be established. By analysing the evoked ERP components, the visual effect can be measured and compared between different patterns, from which we can determine the effect of pattern change on viewers’ visual response. Firstly, the visual response to each patterned appearance is measured, and the responses of the two paired patterned appearances are compared by analysing the evoked ERP components. Statistical technique: hypothesis test and confidence interval estimation are used to calculate the difference in the amplitude and latency of the ERP components; and hence, to determine the different visual effect trigged by the pattern changes of the fabric.

### 2.3. EEG Experiment

#### 2.3.1. Participants

Twenty participants; 9 women and 11 men, between 23 and 54 years of age, took part in this experiment. Their mean age was 31.6 years old with 9.0 years standard deviation. All participants had no known mental problems neither any brain operation nor suffering from epilepsy or claustrophobia. All participants had normal-or corrected vision by spectacles and were right-handed. Before the experiment, each participant was explained the experimental procedure without revealing any detail of the experimental target. The ethical procedure was followed by declaring the procedure and obtaining from each participant written consent and approved before starting of the experiment in line with standard procedure. Each participant was alone and there was not possible for any participant to discuss their experience to another.

#### 2.3.2. EEG Experiment Procedure

**Figure 7:**
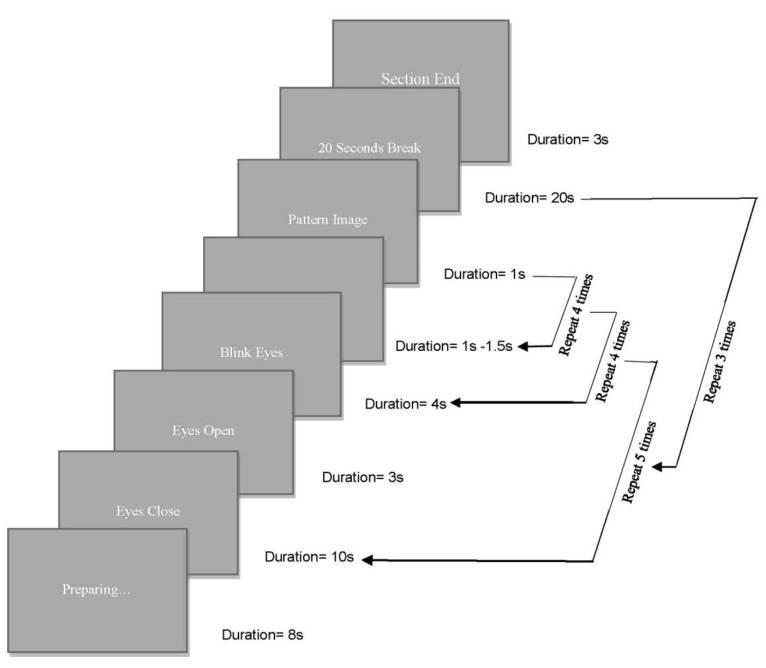
Diagram of the slides with the timing and repeating circles used in the experiment.

The two patterns from every of the four fabrics were scanned, and their clear digital image was saved in the PC. During the experiment each fabric pattern was presented to each participant in a 19-inch screen monitor of a grey background. All pattern images were 305 mm in width and 245 mm in height and shown at the same brightness settings. The sitting distance of every participant from the PC monitor was 1400 mm and the visual angle of stimulation of every pattern in the experiment was 10.0 degrees in the vertical and 12.4 degrees in the horizontal coordinate, and the laboratory was equipped with sound-attenuation. Prior to starting the experimental procedure every participant was given a short explanation of the experiment and were reminded the information on the consent form. An Electro-Cap ECI was placed on the head of the participant in order to record their EEG. A pair of electrodes was used to detect eye movement and one is placed 1 cm above and laterally of the corner of the left eye and the other on the left-hand mastoid behind the ear and lower to the skull. Another electrode was placed on the left earlobe of the participant. The ECI was then connected with the EEC system, and the impedance of all electrodes was checked to be less than 20 kΩ prior to starting the experiment, ensuring their proper attachment to scalp for achieving EEG brain wave data acquisition. A PC displayed the fabric patterns having devised a protocol of slides which had the stimulus of each pattern together with specific instructions. In this protocol, the ‘presentation’ software was used to pre-program in self-written scripts the displaying order and duration of every slide, as shown in Figure 7. At the beginning of the experiment for 8 s a preparatory instruction slide was shown with eye movement commands; ‘eyes close’, ‘eyes open’, and ‘blink eyes’ to relax the eyes of every participant throughout the experiment. A grey screen was then shown for 1.5 s which was the baseline experimental period prior to showing the first pattern for 1 s duration. The repetition of the circles is as shown as in the figure. There is 20 s break between the circles. The pattern stimuli were shuffled in random at the beginning of the presentation and each of them was presented 30 times.

During the experiment, every participant sits on a comfortable armchair opposite the PC monitor with both hands on the arms of the chair, making sure that their eyes were in line with the centre of the monitor. Every participant was informed that a series of slides with fabric patterns will be shown on the monitor and that some of them will give them instructions of closing, opening, or blinking their eyes. Participants were asked to sit comfortably, to be relaxed and to follow the instructions appeared on screen. The operator of the experiment asked each participant to concentrate at the patterns over the period that they appear on the screen without blinking, breathing deep, or moving, and a couple of training familiarity trials were performed by every participant prior to starting the experiment, so that instructions were understood and feeling comfortable. At the end of the experiment the electrodes and the cap were removed from the participants head, the participant was then thanked and asked not to discuss the procedure with anyone else to avoid influencing other participants.

#### 2.3.3. Brain Data Acquisition and Processing

**Figure 8:**
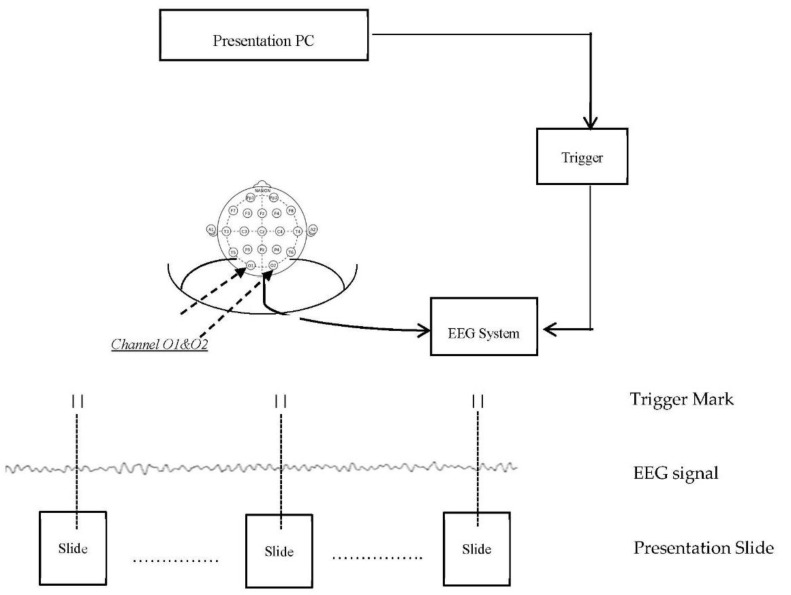
Setup of the EEG signal acquisition in the experiment.

The cap consisting of 19 electrodes was worn on the head of each participant so that their EEG signals could be acquired to determine their brain response to each of the 4 paired fabric patterns. The specific locality of the electrodes on the scalp were according the international 10–20 EEG system [25]. The reference electrode is attached on the left earlobe of each participant and the ground electrode is located at the front of channel Fz. The impedance of the electrodes was less than 20 kΩ. Figure 8 shows the whole set up which consists of the EEG system integrated with the presentation slides in the PC through a trigger box, which places an event marker in the EEG signal for every slide displayed on screen. This enables every slide location along the signal to be known marking any changes in brain waves to each slide stimulation. The electric potential generated by the participant’s eyes movement, named electrooculography (EOG), was also recorded from active and reference bipolar electrodes.

During signal pre-processing, the recordings of the signals of the EEG data and the log file of the presentation slides were inputted to MATLABs EEGLAB (version 11.0.4.3b) toolbox [26], Then, the data recorded from O1 and O2 electrode channels, which are located in the visual brain (see Figure 5) are selected; and the EEG signals that correspond to the eight patterns were extracted. Each signal contained an epoch starting 100 ms prior to pattern stimulus onset until after 1000 ms. There are 30 EEG epochs corresponding to each fabric stimulus. Any artefact caused by eyes blink, movement, temporal muscle activity, or line noise was extracted and cleaned using the Independent Component Analysis (ICA) routine of MATLABs EEGLAB [27]. The artefact-free EEG epochs were then gathered in a STUDY dataset of the EEGLAB to generate the grand average ERP waves that corresponds to the viewing of each pattern stimulus.

## 3. Results

The grand average visual ERPs evoked by the viewing of the patterns are analysed in pairs, which are patterns 1a,b, patterns 2a,b, pattern 3a,b, and patterns 4a,b. In each pair, the ERP waves of channels O1 and O2 are presented separately in two graphs as seen in the following section. The waves start at 100 ms before the pattern onset until after 1000 ms. Vertical grey lines in the graphs indicate the significant differences in between the two ERP waves evoked by the paired patterns at 95% confidence level. The following prominent components were observed in all evoked ERP waves: component P1 at around 100 ms, component N1 at around 150 ms and component P2 between 200 to 300 ms. The amplitude (µv) and latency (ms) of each component were measured. There are two methods to calculate the amplitude of the components N1 and P2 in literature [21]. One is based on the local peak value of the component, and the other one measures the distance between the local peaks of the two successive components: the amplitude of component N1 = P1 peak—N1 peak and the amplitude of component P2 = P2 peak—N1 peak. Both methods have their own strengths and shortcomings. The current experiment used both methods so that one can complement the other. Comparison was conducted on ERP components that were evoked by the two paired patterns of each fabric. The difference of the pattern change response of every participant is calculated by the formula

∑∆n = Pn_1_ − Pn_2_ (1)

where ∑∆n is the different response of pattern change n.

n = 1, 2, 3, 4 and Pn_1_ − Pn_2_ is the response difference of pattern 1 and 2 of each of the four patterned paired fabrics.

Using the statistical hypothesis test (SHT) and confident interval estimation (CIE), the mean of the difference is calculated from the data acquired from the 20 participants. Then the Ryan-Joiner Test at 5% SL was used to test the normal distribution (ND) of the sample data. The significance of the results (over 80%) is reported and discussed further.

### 3.1. Significant Differences in the Visual ERPs Evoked by Pattern 1a,b of Fabric 1

**Figure 9:**
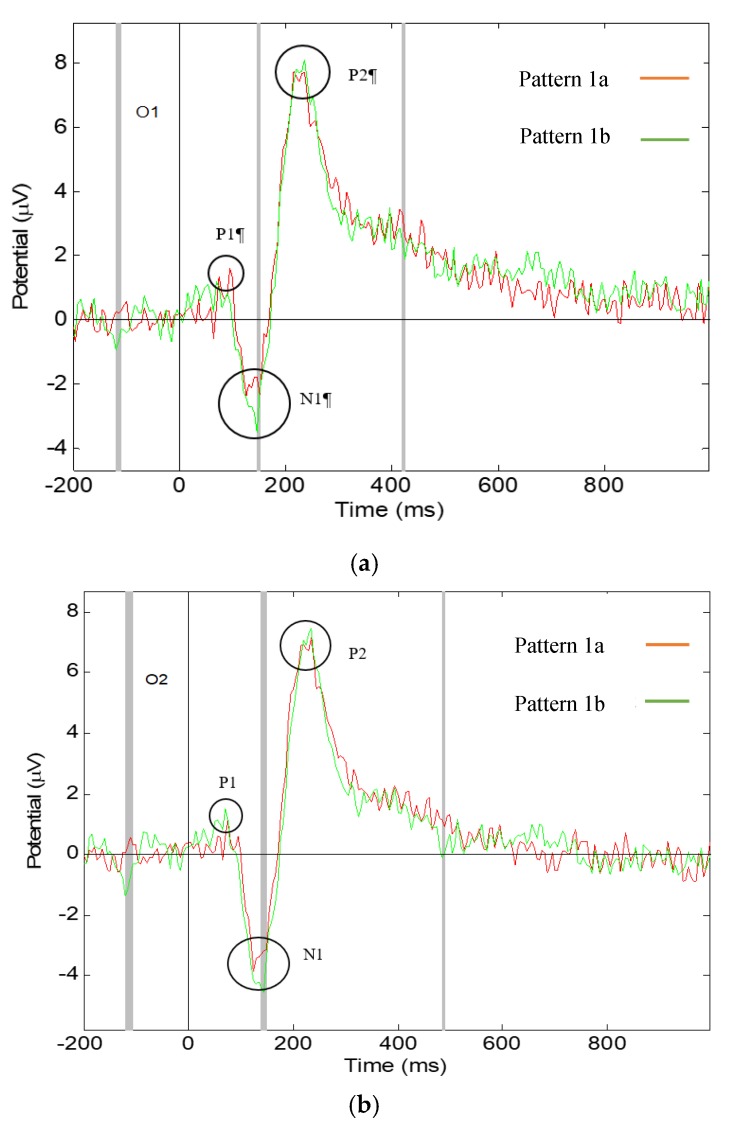
Grand average ERP responding to Pattern 1a,b, the grey lines show the significant difference with *p*-value < 0.05: (**a**) measured at O1 electrode channel; (**b**) measured at O2 electrode channel.

Significant differences have been found in the ERP latency and amplitude components N1 and P2, of Pattern 1a,b, shown in Figure 9, which are reported in Table 1. Pattern 1b triggers an earlier component N1 than Pattern 1a in the left of the visual brain, measured at electrode location O1. Pattern 1b triggers a larger amplitude of component N1 than Pattern 1a, which shows that Pattern 1b has higher visual intensity than Pattern 1a, which is in agreement with another study on amplitude and brightness [28]. In component P2, significant difference was found in the latency in the O2 channel location meaning that Pattern 1a triggers an earlier P2 than Pattern 1b in the right side of the visual brain.

These results show that the difference in intensity of the fabric patterns causes different visual brain responses, in which clearer and well-defined patterns evoke an earlier and larger ERP component N1 and faint patterns evoke an earlier P2 component.

### 3.2. Significant Differences in the Visual ERPs Evoked by Pattern 2a,b of Fabric 2

**Figure 10:**
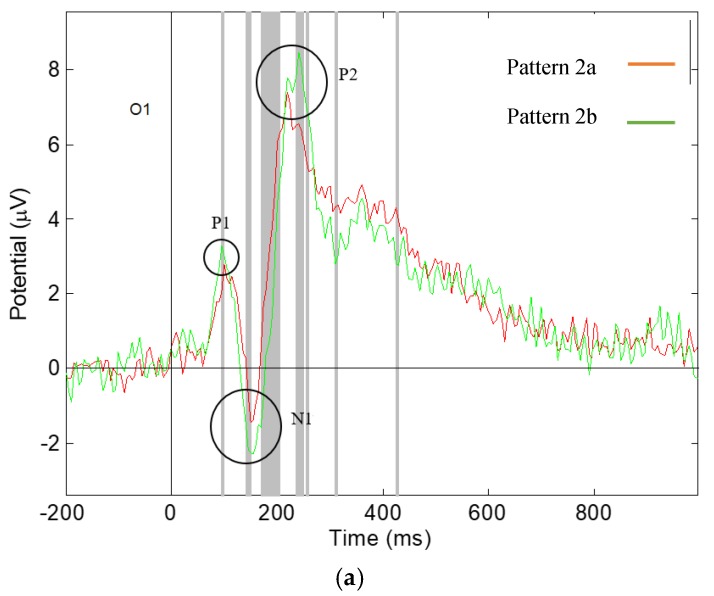

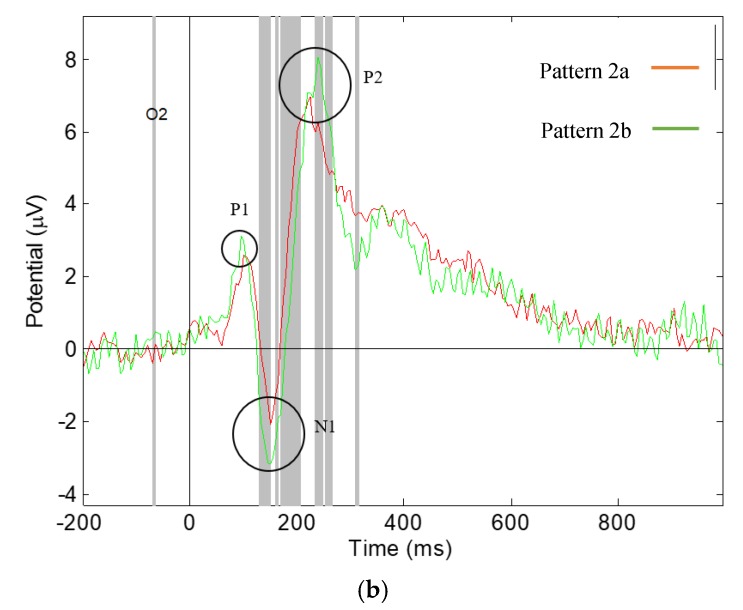
Grand average ERP responding to Pattern 2a,b, the grey lines show the significant difference with *p*-value < 0.05: (**a**) Measured at O1 electrode channel; (**b**) measured at O2 electrode channel.

Significant differences have been found in the amplitude and latency of the ERP components N1 and P2, of Pattern 2a,b, shown in Figure 10, which are reported in Table 2. It was found that there are significant differences in the amplitude of component N1 in both O1 and O2 electrode channels meaning mean Pattern 2b evokes a larger component N1 than Pattern 2a on the both sides of the visual brain, in agreement with other work [19]. In component P2, significant difference was found in the amplitude in both O1 and O2 channel locations, showing that Pattern 2b evokes a larger component P2 than Pattern 2a on both sides of the visual brain. A difference was also found in the latency of component P2 in the O2 brain location, which shows that Pattern 2a gives an earlier component P2 in the right of the visual brain.

The results reveal that pattern changing effects 2a and b of fabric 2 affect the visual response of the participants. The two patterns have symmetrical structures of regular diamond shapes, with Pattern 2a having smaller diamonds to Pattern 2b. The intense effect produced by changing the size of the elements in a pattern causes different responses of the visual brain, in which more intense patterns trigger larger components N1 and P2, indicating a larger brain response.

### 3.3. Significant Differences in the Visual ERPs Evoked by Pattern 3a,b of Fabric 3

**Figure 11:**
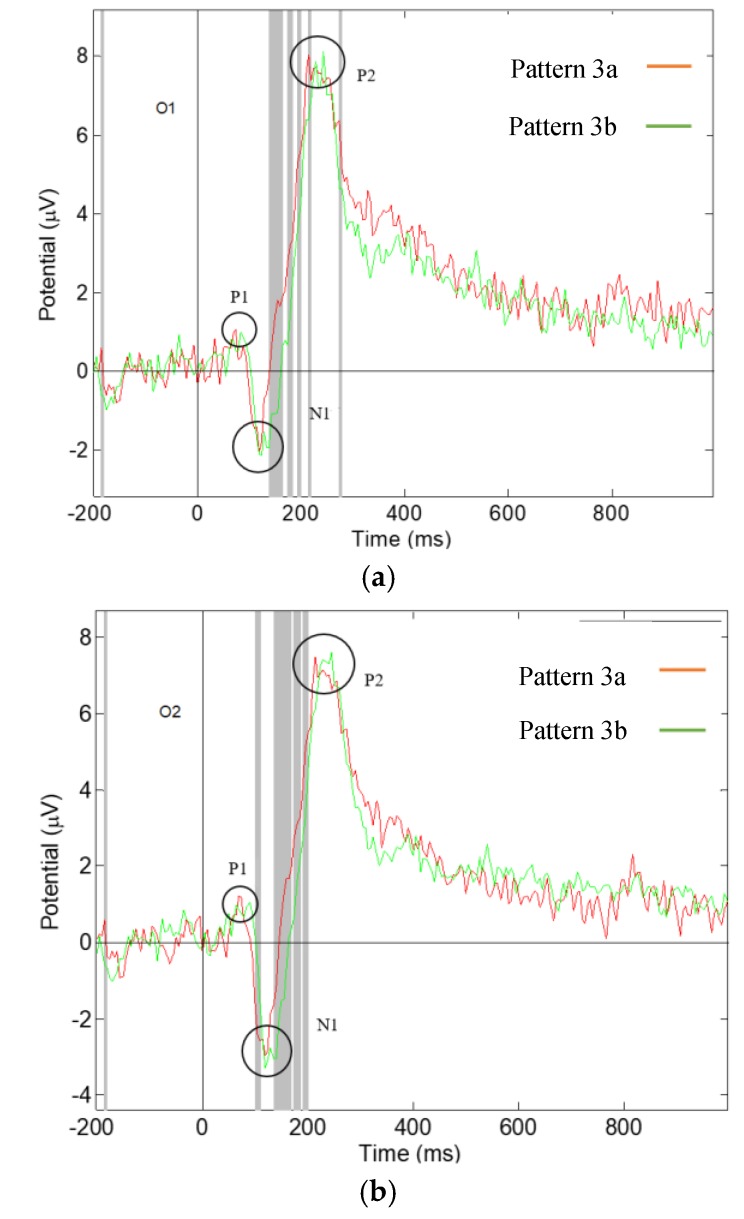
Grand average ERP responding to Pattern 3a,b, the grey lines show the significant difference with *p*-value < 0.05: (**a**) measured at O1 electrode channel; (**b**) measured at O2 electrode channel.

Significant differences have been found in the latency and amplitude of components P1, N1, and P2, of Pattern 3a,b of fabric 3, Figure 11, which are reported in Table 3. In component P1, significant difference was found in the amplitude in the O1 brain location, showing that Pattern 3b evoked a larger component P1 than Pattern 3a in the left of the visual brain. In component N1, significant difference was found in amplitude in both the O1 and O2 channel locations, showing that Pattern 3a evoked a larger component N1 than Pattern 3b in both sides of the visual brain. Significant difference was also found in the latency of component N1 in the O1 channel location, indicating that Pattern 3a evoked an earlier component N1 than Pattern 3b in the left of the visual brain. Our results are in agreement with another study found that the latency of component N1 has been found to be shorter in response to the stimuli with higher brightness [28] and the amplitude of component N1 to be influenced by visual stimuli [19]. The current results reveal different visual parameters between Pattern 3a,b, in which Pattern 3a might have higher visual intensity than Pattern 3b. In component P2, a significant difference was found in the amplitude in the O1 channel location, meaning that Pattern 3b evoked a higher component P2 than Pattern 3a in the left of the visual brain.

The results reveal that our participants were influenced by fabric 3 Pattern 3a,b triggering different responses in the visual brain. Both patterns contain small square shapes of the same size. Pattern 3a has a symmetrical structure of repeating square shapes, whilst Pattern 3b is having asymmetrical randomly arranged squares and rectangular shapes, some filled with intense black colour. Symmetrical patterns with regular repeating elements trigger larger and earlier ERP component N1, indicating a larger and earlier brain response.

### 3.4. Significant Differences in the Visual ERPs Evoked by Pattern 4a,b of Fabric 4

**Figure 12:**
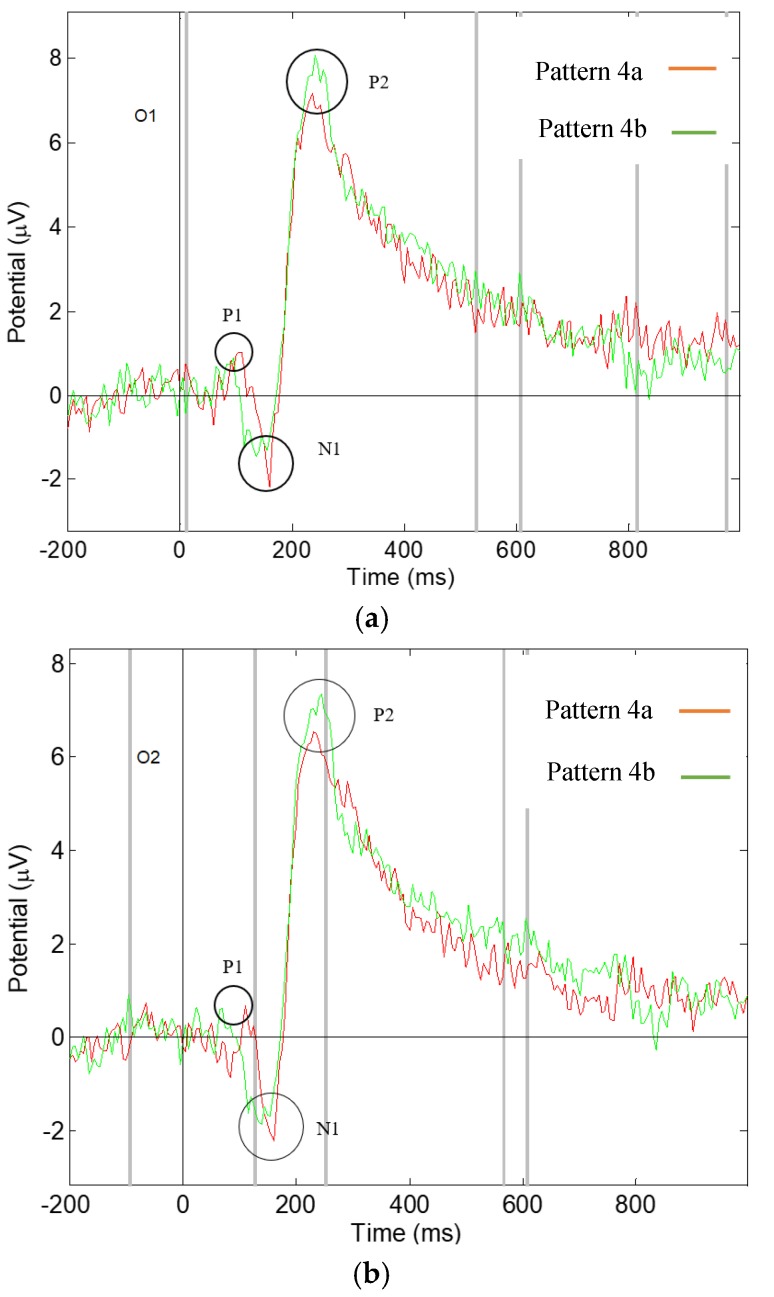
Grand average ERP responding to Pattern 4a,b, the grey lines show the significant difference with *p*-value < 0.05: (**a**) measured at O1 electrode channel; (**b**) measured at O2 electrode channel.

Significant difference was found in the component P1, N1, and P2 on the O1 channel and in components N1 and P2 on the O2 channel, of Pattern 4a,b, fabric 4, Figure 12, as shown in Table 4. In component P1, significant difference was found in latency in the O1 channel location, indicating that Pattern 4a produced an earlier component P1 than Pattern 4b in the left of the visual brain. In component N1, significant difference was found in amplitude in channel O1 location, showing that Pattern 4a evoked a larger component N1 than Pattern 4b in the left of the visual brain, and significant difference was also found in the latency of component N1 in both the O1 and O2 brain locations, indicating that Pattern 4b evoked an earlier component N1 than Pattern 4a in both sides of the visual brain, in agreement with other work [19,28]. It was also found that component P2 had significant difference in the amplitude of both the O1 and O2 channel locations, and in latency at O1 channel. indicating that Pattern 4b produced a larger component P2 than Pattern 4a in both sides of the visual brain, and latency indicating that Pattern 4b triggers an earlier component P2 than Pattern 4a in the left of the visual brain.

The results reveal that the visual brain of our participants was influenced by fabric 4 in different ways. The patterns of fabric 4 contain large square shapes, with Pattern 4a having symmetrical structure of regularly repeating square shapes, whilst Pattern 4b having the same square shapes but non-repeating with some filled with black colour and also having smaller squares within larger ones. Consequently, Pattern 4b is more complex and more asymmetrical than Pattern 4a. The results reveal that these differences trigger different responses in the visual brain, in which the relatively simple and symmetrical patterns trigger an earlier component P1 and a larger component N1, indicating an earlier and larger brain response.

In summary the pattern-changing effect of fabrics 1 and 2 produce a larger N1 response in the visual brain because its pattern changes from a light and loose to a darker and higher contrast effect. However, this phenomenon does not apply in the case of Fabric 3, in which, the symmetrical structure of this pattern evokes a higher response in the visual brain. The same result is observed in the pattern-changing effect of Fabric 4. These results are in agreement with the findings of symmetry/asymmetry studies in literature [16], in which the symmetrical patterns have been found to be detected and processed more easily by the human visual system in comparison with the asymmetrical patterns, as well as with a study of pattern intensity in amplitude and latency of the visual brain [19,28]. This could have a direct connection with pleasure and happiness found in complimentary study.

## 4. Discussion and Conclusions

This study has investigated the influences caused by pattern-changing of four pairs of colourless SMART fabrics on viewer’s visual response using the ERP technique. This was possible by developing a novel composite electrochromic yarn of 20 tex cotton wrapped around silver-plate 0.10 mm diameter copper wire in a Gemmill and Dunsmore hollow spindle fancy yarn machine and successfully functionalised using the Chromazon’s Sprayable System by a pigment which enables it to activate at 31 °C, in less than 40 s. The yarn was subsequently knitted into four fabric pattern pairs testing geometry, asymmetry, and intensity, of our visual brain, at O1 and O2 electrodes positioned at the back of our brain. The responses of 20 participants were analysed by comparing the amplitude and latency components N1, P1, and P2 in the elicited ERP wave from the visual brain. Our results found significant differences in visual brain responses. These differences were mainly observed in components N1, P1, and P2 caused by the difference of the visual parameters of the fabric stimuli. The properties of N1 and P1 components are regarded as exogenous i.e., of pure sensory processing [29]. This process takes place at about 100 ms from the presentation of the stimulus and is said to be influenced by the physical properties of the stimulus. However, there are some researchers that believe that these components could be endogenous and hence could be enhanced by attention [30]. Although there is a general agreement that the P2 component is associated with memory updating, we will take the view that stimulus is processed in a number of either parallel or hierarchical stages which include pattern encoding, recognition, classification, task evaluation followed by response selection and execution. In summary, there must be two stages; a neutral detection of pattern and a make use of this pattern, recognising that some parallel processing takes place, we deal more with the former in our case. Our findings show that the pattern-changing effect evokes different visual responses in component N1. The pattern-changing effect of fabrics 1 and 2 produce a larger N1 response in the visual brain because its pattern changes from a light and loose to a darker and higher contrast effect. However, this phenomenon does not apply in the case of Fabric 3, in which, the symmetrical structure of this pattern evokes a higher response in the visual brain. The same result is observed in the pattern-changing effect of Fabric 4. These results are in agreement with the findings of symmetry/asymmetry studies in literature [16], in which the symmetrical patterns have been found to be detected and processed more easily by the human visual system in comparison with the asymmetrical patterns, and it is said to be associated with pleasure and happiness. Our point to our findings is challenging our current knowledge on perception. We claim that since N1 and P2 are very early in the visual process and the brain is yet to complete perception, i.e., grouping the images together and separating them from one another. Why have our paired patterns have been separated by stronger N1 for the symmetrical and well defined Patterns 1b and 2b—against their complimentary but nevertheless faint Patterns 1a and 2a? Why do we produce stronger N1 in high intensity and symmetrical Patterns 3a and 4a against their non symmetrical counterparts 3b and 4b? These questions may be supported by the physical study of the eye, which indicate that the primary visual cortex is densely populated with layers of cells which prefer stimuli in the shape of bars or angles of an orientation and direction. With our results one can speculate that although the visual processing mechanisms are not yet completely understood, during the information process which is about shape and colourless (with no movement and no spatial organisation) there is a kind of conditioning of the initialisation of this process that may influence our preferences and emotions, which are detectable in other parts of the brain [17], but are outside the scope of this paper. The results of these experiments have high confidence level, which show that different patterns and pattern-changing effects are highly significant. Whether exogenous or endogenous the question is what is the influence of patterns during the initial processing in the brain and since our results show consistency, does this mean that we can use this knowledge to design materials or to do art? The conclusion of this research is that our brain reacts consistently different to things that is sees. Object details as seen in the case of patterns are important to our brain; the SMART fabrics demonstrate this very well by changing from one pattern to an opposite testing this hypothesis. The consequence is that since we now know what object parameters precisely affect our visual brain and its processing, we are not restricted to engineering this change in objects to textile patterns but can apply this knowledge to the design of any objects. In this context, SMART fabrics can switch emotions as they can change from one pattern to another. These SMART textiles we name ‘psychotextiles’ because they are purposely designed to change a specific brain emotion. The same concept can be extended to colour, to shape, to touch, and to taste. Finally, this study, however restricted in size poses a philosophical fundamental question: if these consistent visual brain responses are evoked in less than 200 ms, knowing that brain processing is not complete, does this mean that our ‘primitive world’ is predetermined in our minds, as Aristotelean forms, which get enhanced by memory through perception and experience?

## Figures and Tables

**Table 1:** Amplitude and latency differences of N and P components of visual ERP waves evoked by Pattern 1a,b

O1 Electrode Channel.								
N1	Latency (ms)	N	Mean	StDev	SE Mean	90% CI	T	*p*
		20	4.5	9.99	2.23	(0.64, 8.36)	2.02	0.058
**O2 Electrode Channel**								
N1	Amplitude (µv)	N	Mean	St Dev	SE Mean	90% CI	T	*p*
		20	1.023	2.527	0.565	(0.046, 2.000)	1.81	0.086
P2	Latency (ms)	N	Mean	St Dev	SE Mean	90% CI	T	*p*
		20	−9.5	12.97	2.9	(−14.51, −4.49)	−3.28	0.004

St Dev: Standard deviation. SE Mean: Standard error of the mean. CI: Confidence interval. *p*: *p*-value.

**Table 2:** Amplitude and latency differences of N and P components of visual ERP waves evoked by Pattern 2a,b.

O1 Electrode Channel								
N1	Amplitude (µv)	N	Mean	St Dev	SE Mean	95% CI	T	*p*
		20	2.143	2.662	0.595	(0.898, 3.389)	3.6	0.002
P2	Amplitude (µv)	N	Mean	St Dev	SE Mean	95% CI	T	*p*
		20	−3.024	3.271	0.731	(−4.555, −1.493)	−4.13	0.001
**O2 Electrode Channel**								
N1	Amplitude (µv)	N	Mean	St Dev	SE Mean	98% CI	T	*p*
		20	2.201	2.469	0.552	(0.799, 3.603)	3.99	0.001
P2	Amplitude (µv)	N	Mean	St Dev	SE Mean	95% CI	T	*p*
		20	−2.943	3.048	0.681	(−4.369, −1.516)	−4.32	0
	Latency (ms)	N	Mean	St Dev	SE Mean	95% CI	T	*p*
		20	−7.75	10.82	2.42	(−12.81, −2.69)	−3.2	0.005

St Dev: Standard deviation. SE Mean: Standard error of the mean. CI: Confidence interval. *p*: *p*-value.

**Table 3:** Amplitude and latency differences of N and P components of visual ERP waves evoked by Pattern 3a,b.

O1 Electrode Channel								
P1	Amplitude (µv)	N	Mean	St Dev	SE Mean	85% CI	T	*p*
		20	−0.905	2.641	0.591	(−1.791, −0.019)	−1.53	0.142
N1	Amplitude (µv)	N	Mean	St Dev	SE Mean	80% CI	T	*p*
		20	−1.333	4.044	0.904	(−2.534, −0.133)	−1.47	0.157
	Latency (ms)	N	Mean	St Dev	SE Mean	89% CI	T	*p*
		19	−3.68	9.4	2.16	(−7.31, −0.06)	−1.71	0.105
P2	Amplitude (µv)	N	Mean	St Dev	SE Mean	85% CI	T	*p*
		18	−0.645	1.785	0.421	(−1.279, −0.011)	−1.53	0.143
**O2 Electrode Channel**								
N1	Amplitude (µv)	N	Mean	St Dev	SE Mean	83% CI	T	*p*
		20	−1.074	3.319	0.742	(−2.132, −0.015)	−1.45	0.164

St Dev: Standard deviation. SE Mean: Standard error of the mean. CI: Confidence interval. *p*: *p*-value.

**Table 4:** Amplitude and latency differences of N and P components of visual ERP waves evoked by Pattern 4a,b.

O1 Electrode Channel								
P1	Latency (ms)	N	Mean	St Dev	SE Mean	90% CI	T	p
		18	−8.06	16.37	3.86	(−14.77, −1.34)	−2.09	0.052
N1	Amplitude (µv)	N	Mean	St Dev	SE Mean	95% CI	T	p
		17	−11.33	5.91	1.43	(−14.37, −8.29)	−7.91	0
	Latency (ms)	N	Mean	St Dev	SE Mean	95% CI	T	p
		18	10.28	17.1	4.03	(1.77, 18.78)	2.55	0.021
P2	Amplitude (µv)	N	Mean	St Dev	SE Mean	95% CI	T	p
		17	−1.934	2.992	0.726	(−3.473, −0.396)	−2.67	0.017
	Latency (ms)	N	Mean	St Dev	SE Mean	80% CI	T	p
		19	3.95	11.74	2.69	(0.37, 7.53)	1.47	0.16
**O2 Electrode Channel**								
N1	Latency (ms)	N	Mean	St Dev	SE Mean	88% CI	T	p
		20	5.5	14.95	3.34	(0.06, 10.94)	1.65	0.116
P2	Amplitude (µv)	N	Mean	St Dev	SE Mean	91% CI	T	p
		20	−1.253	3.093	0.692	(−2.488, −0.017)	−1.81	0.086

St Dev: Standard deviation. SE Mean: Standard error of the mean. CI: Confidence interval. *p*: *p*-value.
